# Exploring the impact of breast cancer on colonization resistance of mouse microbiota using network node manipulation

**DOI:** 10.1016/j.heliyon.2024.e30914

**Published:** 2024-05-11

**Authors:** Alejandra Wu-Chuang, Lourdes Mateos-Hernandez, Lianet Abuin-Denis, Apolline Maitre, Janet Avellanet, Arlem García, Dasha Fuentes, Alejandro Cabezas-Cruz

**Affiliations:** a Anses, INRAE, Ecole Nationale Vétérinaire d’Alfort, UMR BIPAR, Laboratoire de Santé Animale, Maisons-Alfort, F-94700, France; b Animal Biotechnology Department, Center for Genetic Engineering and Biotechnology, Avenue 31 between 158 and 190, P.O. Box 6162, 10600, Havana, Cuba; c INRAE, UR 0045 Laboratoire de Recherches Sur Le Développement de L'Elevage (SELMET-LRDE), Corte, France; d EA 7310, Laboratoire de Virologie, Université de Corse, Corte, France; e Center of Molecular Immunology (CIM), Calle 15 esq. 216, Atabey, Playa, Havana, Cuba; f National Center for Laboratory Animal Breeding (CENPALAB), Calle 3ra # 40759 entre 6ta y carretera de Tirabeque, Rpto La Unión, Boyeros, Havana, Cuba

**Keywords:** Breast cancer, Gut microbiome

## Abstract

Breast cancer, a global health concern affecting women, has been linked to alterations in the gut microbiota, impacting various aspects of human health. This study investigates the interplay between breast cancer and the gut microbiome, particularly focusing on colonization resistance—an essential feature of the microbiota's ability to prevent pathogenic overgrowth. Using a mouse model of breast cancer, we employ diversity analysis, co-occurrence network analysis, and robustness tests to elucidate the impact of breast cancer on microbiome dynamics. Our results reveal that breast cancer exposure affects the bacterial community's composition and structure, with temporal dynamics playing a role. Network analysis demonstrates that breast cancer disrupts microbial interactions and decreases network complexity, potentially compromising colonization resistance. Moreover, network robustness analysis shows the susceptibility of the microbiota to node removal, indicating potential vulnerability to pathogenic colonization. Additionally, predicted metabolic profiling of the microbiome highlights the significance of the enzyme EC 6.2.1.2 - Butyrate--CoA ligase, potentially increasing butyrate, and balancing the reduction of colonization resistance. The identification of *Rubrobacter* as a key contributor to this enzyme suggests its role in shaping the microbiota's response to breast cancer. This study uncovers the intricate relationship between breast cancer, the gut microbiome, and colonization resistance, providing insights into potential therapeutic strategies and diagnostic approaches for breast cancer patients.

## List of abbreviations

ALDEx2: ANOVA-like differential expression 2
ASVs: amplicon sequence variants
APL: Average Path Length
Clr: centered log-ratio
COGs: Cluster of Orthologous Genes
Dpi: Days post-inoculation
EC: Enzyme Classification numbers
KEGG: Kyoto Encyclopedia of Genes and Genomes
LCC: Largest Connected Component
CENPALAB: National Center for Laboratory Animal Breeding
NEC: Neutropenic enterocolitis
NSTI: nearest sequenced taxon index
PBS: phosphate-buffered saline
PICRUSt2: phylogenetic Investigation of Communities by Reconstruction of Unobserved States 2
QIIME: Quantitative Into Microbial Ecology
SparCC: Sparse Correlations for compositional data
SPF: specific pathogens free
SRA: Sequence Read Archive

## 1 Introduction

Breast cancer, a prevalent malignancy affecting women worldwide, continues to be a significant global health concern [1]. Recent research has unveiled the pivotal role of the gut microbiota in diverse aspects of human health, including cancer development and progression [2]. Understanding the complex interplay between breast cancer and the gut microbiome holds great promise for potential therapeutic interventions and diagnostic approaches. In human studies, researchers have observed distinct microbial signatures in the gut microbiota of breast cancer patients compared to healthy individuals [[3], [4], [5]]. These alterations often involve changes in the relative abundance of specific bacterial taxa. For instance, decreased levels of beneficial bacteria such as *Bifidobacterium* and *Lactobacillus*, along with an increase in potentially harmful bacteria like *Escherichia coli* and *Fusobacterium*, have been reported in breast cancer patients [6]. These shifts in microbial composition have been associated with inflammation, immune dysregulation, and the promotion of tumor growth [7]. Similarly, mouse models have been instrumental in elucidating the causal relationships between breast cancer and gut microbiota. Studies using various mouse models of breast cancer have shown that tumor development can lead to significant changes in the gut microbial community. For instance, tumor growth has been associated with reduced microbial diversity and altered bacterial composition, similar to what has been observed in human patients.

Network analysis is a powerful tool that allows researchers to investigate the complex interactions and associations between different microbial taxa within a community [8,9]. By constructing co-occurrence networks based on correlations between microbial abundances, researchers can gain insights into the structure, organization, and stability of the microbial community [10,11]. In the context of cancer, network analysis has been applied to study the changes in the microbiota and its interactions following exposure to cancer cells [12,13]. By examining the co-occurrence patterns of bacterial taxa, researchers can identify potential keystone taxa—microbes that play critical roles in maintaining the stability and functioning of the microbial community [[14], [15], [16]]. These keystone taxa can serve as important indicators of changes in the microbiota's structure and function due to cancer [17]. For instance, some studies have shown that breast cancer can lead to a reduction in microbial diversity and complexity, resulting in a less connected and stable microbial network [18]. Other studies have identified specific keystone taxa that show differential abundance or centrality in the microbial networks of breast cancer-bearing mice compared to healthy controls [18]. These keystone taxa may have crucial roles in shaping the gut microenvironment and influencing host-microbe interactions.

Despite these notable advancements, an important aspect remains unexplored in the context of breast cancer and the gut microbiome: the impact of breast cancer on colonization resistance. Colonization resistance refers to the ability of the resident gut microbiota to prevent the establishment of potentially harmful or pathogenic microorganisms in the gut [[19], [20], [21], [22]]. Although this phenomenon has been extensively studied in the context of various diseases and conditions [[19], [20], [21], [22]], its specific association with breast cancer remains largely uncharted territory.

Additionally, while there is a significant body of evidence highlighting the increased susceptibility of cancer patients to various infectious diseases, including those involving the intestinal tract [23], the role of cancer, particularly breast cancer, in relation to colonization resistance remains unexplored. The vulnerability of cancer patients to infections, spanning bacterial [24], fungal [25], and viral [26] origins, is attributed to both cancer-driven factors and the unintended consequences of intensive therapeutic regimens [27]. The intricate web of mechanisms that leads to immune compromise in cancer patients, regardless of cancer type, and contributes to the heightened frequency and severity of infectious complications in this population is multifaceted and interconnected. Yet, the pivotal role of cancer, particularly breast cancer, in shaping colonization resistance, which is crucial for preventing the overgrowth of harmful microbes in the gut, has remained conspicuously unaddressed.

In this study, we aim to bridge this knowledge gap by conducting a comprehensive analysis of the gut microbiota in a mouse model of breast cancer. By focusing on colonization resistance, we seek to investigate how breast cancer influences the gut microbiome's ability to resist colonization by pathogenic microorganisms. To achieve this, we utilized network analysis to examine the co-occurrence patterns and interactions between microbial taxa in the gut microbiota of breast cancer-bearing mice. In addition to network analysis, we employed network robustness analysis using node removal and node addition methods to further elucidate the role of colonization resistance in the context of breast cancer and the gut microbiome. To test colonization resistance using network robustness, one approach is to simulate the removal [9,28] or addition of nodes (microbes) [29] in the network and observe the impact on the overall stability and functionality of the community. If the resident microbial network exhibits high robustness, it suggests that the community is more resistant to the colonization or invasion by pathogens. By unraveling the intricacies of colonization resistance in the context of breast cancer, our study aims to contribute novel insights into the interplay between breast cancer progression and the gut microbiome.

## 2 Materials and methods

### 2.1 Mice and housing conditions

Female BALB/c/Cenp specific pathogens free (SPF) mice aged 6–8 weeks were sourced from CENPALAB (Havana, Cuba) and housed in standard Tecniplast cages (Varese, Italy). They had *ad libitum* access to autoclaved EAO 1004 food (CENPALAB, Havana, Cuba) and water. The housing conditions included controlled room temperature (20–23 °C), humidity (65 ± 10 %), and a 12-h light-dark cycle regulated automatically. An experienced technician performed twice-daily monitoring of the mice for any abnormal reactions, health issues, or complications.

### 2.2 Tumor cell line

The stocks of triple-negative mouse breast cancer 4T1 cells were cultured in DMEM/F12 medium (Gibco BRL, Grand Island, NY, USA) supplemented with 10 % fetal bovine serum (Gibco BRL, Grand Island, NY, USA). Cell viability was assessed using the trypan blue exclusion assay. For inoculation, 1 × 10^5^ 4T1 cells were diluted in 1 mL of phosphate-buffered saline (PBS, CENPALAB, Havana, Cuba).

### 2.3 4T1 tumor cells inoculation

BALB/c mice were subcutaneously inoculated with 100 μL of 4T1 tumor cells (1 × 10^4^ cells/mouse) into the fourth mammary fat pad. Tumor implantation was monitored by gently palpating the tumor inoculation site every alternate day. Following inoculation, tumor size was measured twice a week using a caliper (Mitutoyo, Kanagawa, Japan). The volume of each individual tumor was calculated using the formula: V = πab^2^/6, where 'a' represents the major tumor diameter and 'b' represents the minor diameter. Mice were euthanized if tumors reached 2000 mm^3^ or became ulcerated, using cervical dislocation to ensure animal welfare. Euthanasia was achieved via cervical dislocation. A group of non-inoculated BALB/c mice was used as the control group.

### 2.4 Evaluation of tumor-associated effects

Clinical signs, symptoms, and the morbidity and mortality of each animal were assessed on a daily basis. Body weight was measured using a precision balance (Sartorius, Germany) at the study's onset and weekly following tumor inoculation. After 23 days of tumor cell inoculation, animals were anesthetized through intraperitoneal administration of a combination of Ketamine/Diazepam/Atropina (50/5/1 mg/kg) (AICA, Havana, Cuba), followed by euthanasia via cervical dislocation. To investigate the invasion of tumor neighboring tissues, tumors were removed.

### 2.5 Mouse feces collection and DNA extraction

Fecal samples were collected on sterile tubes. Fresh feces were collected from each animal at 16 days post-inoculation (dpi) and 22 dpi. Fecal samples were stored in sterile tubes at −20 °C. Fecal genomic DNA was extracted using a Nucleospin tissue DNA extraction Kit (Macherey-Nagel, Hoerdt, France). Each DNA sample was eluted in 100 μl of sterile water. Genomic DNA quality (OD260/280 between 1.8 and 2.0) was measured with NanoDrop™ One (Thermo Scientific, Waltham, MA, USA).

### 2.6 Illumina library preparation and sequencing of the 16S rRNA gene

At least 200 ng of mouse feces DNA at ≥ 20 ng/μL concentration were sent for amplicon sequencing of the bacterial 16S rRNA gene, which was commissioned to Novogene Bioinformatics Technology Co. (London, UK). Libraries were prepared with NEBNext® Ultra™ IIDNA Library Prep Kit (New England Biolabs, MA, USA). A single lane of Illumina MiSeq system was used to generate 251-base paired-end reads from the V4 variable region of the 16S rRNA gene using bar-coded universal primers 515F [30] and 806 R [31] in mouse fecal samples from inoculated (n = 17, 9 samples at 16 dpi and 8 samples at 22 dpi) and non-inoculated (n = 14, 7 samples per time point) mice. The raw 16S rRNA gene sequences obtained from mouse feces samples were deposited at the SRA repository (Bioproject No. PRJNA1008984).

### 2.7 Controls, identification and bioinformatic removal of contaminants

Four extraction reagent controls were performed in which different DNA extraction steps were done using the same conditions as for the samples but using water as template. DNA amplification from the water control samples was performed under the same conditions as for any other sample. Possible contaminating DNA in samples for 16S rRNA gene sequencing was statistically identified with ‘decontam’ package [32], implemented in R, using the ‘prevalence’ method. Prevalence is defined as the presence or absence across the sample and the method used compares the prevalence of each sequence feature in true samples to the prevalence in negative controls, in order to identify contaminants. Then, contaminants were removed from the dataset before downstream microbiome analysis.

### 2.8 Analysis of 16S rRNA gene amplicon sequences

The analysis of 16S rRNA gene sequences was performed using Quantitative Into Microbial Ecology (QIIME) 2 pipeline (v. 2021.4) [33]. Using DADA2 software [34] implemented in QIIME2, 16S rRNA gene sequences were first demultiplexed and then quality trimmed based on the average quality per base of the forward and reverse reads. Consequently, reads were merged and chimeric variants were removed. The resulting representative sequences were taxonomically assigned using a pre-trained naïve Bayes taxonomic classifier [35] based on SILVA database version 132 [36] and the 515F/806 R primer set. The resulting taxonomic data tables were collapsed at genus level and low abundant taxa were removed by filtering taxa with less than 10 total reads and present in less than 30 % of samples. The taxonomic data tables were used for network analysis and keystone taxa identification.

Construction of bacterial co-occurrence networks and identification of keystone taxa.

Co-occurrence network analyses were performed using the Sparse Correlations for compositional data (SparCC) method [37] implemented in R studio [38]. Taxonomic data tables were used to calculate the correlation matrix. Correlation coefficients with magnitude >0.75 or < −0.75 were selected. Network visualization and calculation of topological features and taxa connectedness (i.e., number of nodes and edges, modularity, network diameter, average degree, weighted degree, clustering coefficient and centrality metrics) was performed using the software Gephi 0.9.2 [39]. Core co-occurrence networks were constructed by choosing correlation coefficients with magnitude >0.95 or < −0.95. For keystone taxa identification, three different criteria were used: (i) high eigenvector centrality, which measures the importance of a node in a co-occurrence network while considering the relevance of their neighbors [40] (ii) ubiquitousness (i.e., bacterial taxa present across all the samples at one condition) and (iii) high abundance. Cutoff values of 0.35 and 0.75 were selected for the mean of the abundance and the eigenvector centrality, respectively. Scatter plot was done using the software GraphPad 9 Prism (GraphPad Software Inc., San Diego, CA, USA).

### 2.9 Network robustness analysis

We conducted a thorough evaluation of the robustness of microbial co-occurrence networks by examining how the network connectivity is affected by node removal and addition. To achieve this, we simulated the proportion of node removal required to cause a connectivity loss of 0.80 in each network. The analysis involved using random or directed attacks.

For the directed attack, we employed three strategies: betweenness centrality, degree centrality, and cascading. The betweenness centrality approach involved removing nodes with the highest betweenness centrality values first. In the degree centrality approach, nodes with the highest degree centrality values were removed first. In the cascading approach, nodes with the highest betweenness centrality values were removed first, and after each node removal, betweenness centrality was recalculated.

The network robustness analysis was carried out using the NetSwan package [41] in RStudio [38] (File S1). Additionally, a node addition analysis was performed in RStudio [38], following the method described by Freitas et al. (2020) [29]. In this analysis, new nodes were randomly added to the existing network. Subsequently, we measured two key network metrics: the size of the Largest Connected Component (LCC) and the Average Path Length (APL). The LCC represents the main connected structure of the network, and the APL calculates the average number of steps required to travel between any two nodes in the network, providing an indication of how quickly information can spread through the network. By analyzing both the LCC and APL, we can gain valuable insights into the network's connectivity, robustness, and efficiency. A large LCC and a short APL are generally desirable traits for a well-functioning and effective network.

To ensure accuracy, the simulation was repeated with different sets of nodes, adding 100, 300, 500, 700, and 1000 nodes. The results were visualized using GraphPad Prism 8.0.1, providing a comprehensive understanding of the network's robustness.

### 2.10 Prediction of functional traits in breast cancer-bearing mouse microbiome

For the metabolic profiling of each sample, PICRUSt2 software [42] was used for the prediction of functional gene abundances based on 16S rRNA gene amplicon sequences. Briefly, the amplicon sequence variants (ASVs) were aligned and placed into a reference tree (NSTI cut-off value of 2), which was then used to infer gene family copy numbers of each ASVs and finally determine gene family abundance per sample. Kyoto Encyclopedia of Genes and Genomes (KEGG) orthologs (KO) [43], Enzyme Classification numbers (EC) and Cluster of Orthologous Genes (COGs) [44] were used as gene family catalogues for the predictions. Pathway profiles were inferred from structured pathway mapping based on MetaCyc database [45]. Linkages between ASVs (collapsed at genus level) and predicted functions (pathways) were assessed using the function “Taxa contribution” from PICRUSt2 metagenome predictions.

### 2.11 Statistical analysis

Taxonomic data tables, which consisted of sequencing-read counts, were used as input of the R package ‘ALDEx2’ [46], which performed centered log-ratio (clr) transformation for all features in all the samples and subsequently compared statistically the taxa abundances using Kruskal-Wallis test. Enzyme and pathway abundances were compared using the R package ‘DeSeq2’ [47]. The number of shared taxa and nodes between the different experimental conditions were visualized using UpSet plots [48,49]. The number of shared keystone taxa and enzymes in the different experimental conditions were visualized using Venn diagrams implemented in the online tool http://bioinformatics.psb.ugent.be/webtools/Venn/.

Alpha and beta-diversity of bacterial taxa and functional profile were carried out on rarified ASV and enzyme tables, respectively. Differences in alpha-diversity metrics between groups were tested using a pairwise Kruskal-Wallis test. Beta-diversity of bacterial taxa was explored using the Jaccard and the Weighted Unifrac indexes. Beta-diversity metrics were compared among the groups using a PERMANOVA test. Differences were considered significant when *p* < 0.05.

The analysis of node removal utilized bootstrapping method to calculate confidence intervals. Additionally, the node addition analysis employed Wilcoxon signed-rank tests to assess whether there were significant differences in the mean size of the largest connected component and the average path length from 0. To account for multiple comparisons, the *p*-values from these tests were adjusted using the Benjamini-Hochberg procedure, ensuring robust control over false positives.

## 3 Results

### 3.1 Impact of the exposure to breast cancer cells on the microbiota of mice over the time

**Fig. 1:**
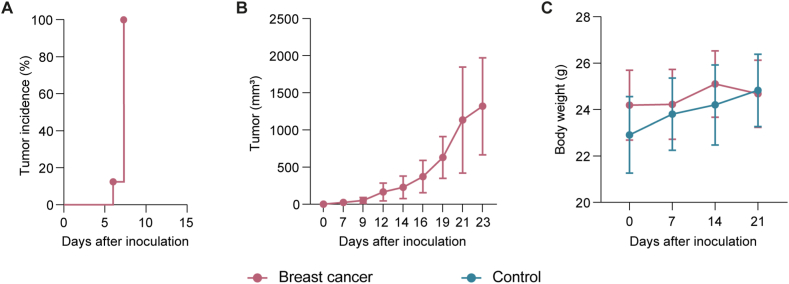
All 4T1 inoculated BALB/c mice developed tumor growth. (A) Latency of tumor cells (B) Growth kinetic of tumor in BALB/c mice. (C) Body weight of control and breast cancer bearing-mice. Results shown represent the means and standard error values. Data were compared using Students t-test. No significant differences (*p* > 0.05) were detected between groups (n = 8).

To study the impact of breast cancer on mouse microbiota, BALB/c mice were inoculated with 4T1 cancer cells. As shown in Fig. 1A and B, all mice developed tumors. 4T1 cells grew by filling the subcutis, although some tumors evidenced signs of active invasion of neighboring tissues, such as muscle and dermis (Table 1). The analysis of body weight of mice did not reveal statistically significant differences (Students *t*-test, *p* > 0.05) between bearing-tumor and control mice (Fig. 1C).

**Table 1:** Local invasiveness of the mouse breast cancer 4T1.

Treatment	Tumor localization positive/total animals (%)	
	Subcutis	Neighboring tissues
4T1	8/8 (100 %)	3/8 (37.5 %)

Mouse feces from breast cancer-exposed mice and non-exposed mice (hereafter referred as control mice) were subsequently collected at 16- and 22-days post-inoculation (dpi) and DNA extraction were performed for 16S rRNA gene profiling to study the microbiota. Statistical identification and removal of contaminating DNA features were performed prior to analysis (Supplementary Table S1).

**Fig. 2:**
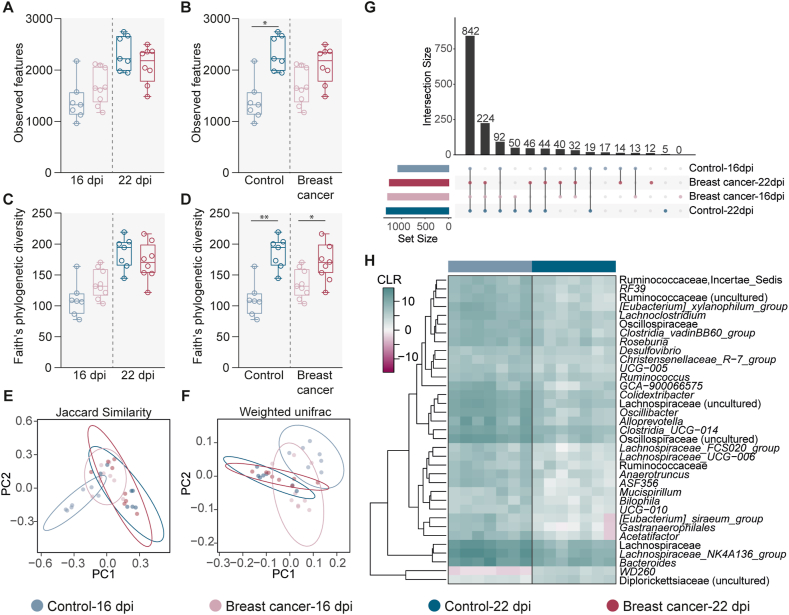
Comparison of the microbial diversity and taxonomic profile of breast cancer-bearing mice at different timepoint or compared to control mice (A, C) Observed features and (B, D) Faith's phylogenetic diversity indexes were used to measure the richness and the biodiversity, respectively, of the microbiota of breast cancer-bearing mice compared to the control one (A, C) or compared to the same group but from different timepoints (B, D) (Kruskal-Wallis, *p < 0.05, **p < 0.01). (E) Jaccard similarity and (F) Weighted Unifrac indexes were used to measure the beta diversity of the microbiota of breast cancer-bearing mice and control one at different timepoints (PERMANOVA, *p < 0.05*). (G) UpSet plot showing the number of common and unique bacterial taxa among the different experimental groups. Each column corresponds to a possible intersection while each row represents a set. Bar chats on the row show the size of each set and bar chats on top show the size of the intersections. The filled-in circles and the connecting lines show which set is part of an intersection. (H) Heatmap representing the abundance (expressed as CLR) of the bacterial taxa that changed significantly between the control groups.

Analysis of alpha diversity indexes showed that the number of bacterial taxa (Fig. 2A) and Faith's phylogenetic diversity (Fig. 2B) did not differ between the control and breast cancer-bearing mice at either 16 or 22 dpi (Kruskal-Wallis, *p >*0.05). However, we did find a significant difference in the number of bacterial taxa in the microbiota of control mice over the course of the study (Fig. 2C, Kruskal-Wallis, *p > 0.05*) which was not the case for breast cancer-exposed mice (Fig. 2C). Moreover, control mice as well as breast cancer-exposed mice showed that the diversity of their microbiota changes significantly over the time (Fig. 2D, Kruskal-Wallis, *p < 0.05*). Beta diversity analysis of mouse microbiota revealed that breast cancer exposure led to a shift in the bacterial community composition and abundance, compared to the control group, as measured using the Jaccard index (PERMANOVA, *F* = 1.69, *p* = 0.003, Fig. 2E) and Weighted unifrac distance (PERMANOVA, *F* = 3.64, *p* = 0.009, Fig. 2F), respectively at 16 dpi (but not at 22 dpi). Beta diversity analysis also revealed that, over the time, the control group as well as breast cancer-bearing mice has a significant difference in the Jaccard index (PERMANOVA, *F* = 1.69, *p* = 0.003, Fig. 2E) and Weighted unifrac distance indexes (PERMANOVA, *F* = 3.64, *p* < 0.01, Fig. 2F).

Comparison of the taxonomic profile between the control and breast cancer-bearing mice at different timepoints showed that a large number of bacteria (i.e., 841/1449) were shared between the different experimental conditions. Furthermore, 17, 5 and 12 bacterial taxa were found exclusively in the microbiota of control groups at 16 dpi, 22 dpi and in breast cancer-bearing mice at 22 dpi, respectively (Fig. 2G–Supplementary Table S2). Pairwise comparisons of the bacterial abundance were also performed between the control and breast cancer-exposed mice, at 16 and 22 dpi, but no significant changes in the abundance of any bacterial taxa were found. Similarly, differential abundance analysis for the microbiota of breast cancer-bearing mice over the time showed no significant differences. However, comparison of the bacterial abundance in the microbiota of control groups at 16 and 22 dpi showed that the abundance of 35 bacterial taxa changed significantly (Fig. 2H, Kruskal-Wallis, *p < 0.05*). These results show that the composition and diversity of the microbiota of breast cancer-bearing mice does not change greatly compared to the control group. However, changes in the composition or diversity of the microbiota over the time can be detected either in the control or breast cancer groups.

### 3.2 Impact of breast cancer on the assembly of mouse microbiota

**Fig. 3:**
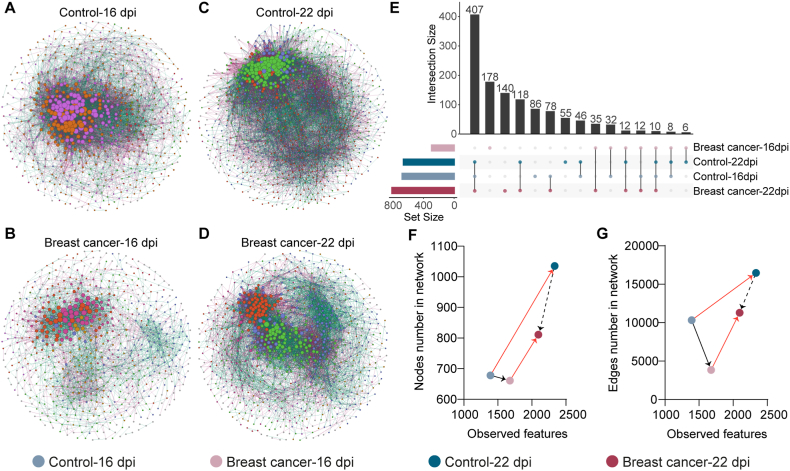
Bacterial community of breast cancer bearing-mice compared to control one at different timepoints. Microbial co-occurrence networks inferred from 16S rRNA gene sequences obtained from (A, C) control mice and (B, D) breast cancer-bearing mice at (A, B) 16 dpi and (C, D) 22 dpi. Nodes represent bacterial taxa and edges stand for a co-occurrence correlation (SparCC >0.75 or < −0.75). Node size is proportional to the eigenvector centrality value and node color is based on the modularity class (nodes with the same color belong to the same cluster). Positive and negative interactions between co-occurring bacteria are represented by the green and dark red edges, respectively. Only nodes with at least one connecting edge are displayed. (E) UpSet plot showing the number of common and unique nodes of microbial co-occurrence networks among the different groups. Each column corresponds to a possible intersection while each row represents a set. Bar chats on the row show the size of each set and bar chats on top show the size of the intersections. The filled-in circles and the connecting lines show which set is part of an intersection. (F,G) Scatter plot showing the mean of observed features versus number of (F) nodes and (G) edges found in the microbial co-occurrence networks of different experimental groups. Black arrows show changes in the state of microbiota between the control and breast cancer groups while red arrows show changes in the microbiota state between the same groups of different timepoints. (For interpretation of the references to color in this figure legend, the reader is referred to the Web version of this article.)

The impact of the exposure to breast cancer cells on the mice bacterial community assembly were inferred by co-occurrence networks. Visual inspection of the networks showed that breast cancer-bearing mice present a shift in the bacterial community assembly patterns compared to the control groups at either timepoint (Fig. 3A–D). Changes in the structure of the microbiota within each group over the time could be also appreciated. Analysis of the topological features of the networks revealed a decreased number of nodes and especially, of edges in the microbiota of breast cancer-bearing mice compared to the control groups (Table 2). Similarly, the modularity and the average degree decreased in the breast cancer-bearing mice compared to the control groups (Table 2); however, the number of modules in the co-occurrence networks increased in mice exposed to cancer cells compared to the control groups (Table 2). Comparison of nodes identity showed that only 9 bacterial taxa were shared in the microbial co-occurrence networks of the different experimental groups (Fig. 3E–Supplementary Table S3). On the other hand, a high number of unique taxa was found in the four experimental groups (i.e., 86, 178, 56 and 140 unique bacterial taxa in the control groups at 16, 22 dpi and breast cancer groups at 16, 22 dpi, respectively) (Fig. 3E–Supplementary Table S3). In order to determine how much of the bacterial diversity was engaged in microbe-microbe interactions, comparison of the observed features versus the number of nodes (Fig. 3F) or edges (Fig. 3G) were performed. We found that microbiota of the breast cancer group at 16 dpi was characterized by an increase in the observed features and decrease in the number of nodes or edges compared to its control group (Fig. 3F and G, black lines). Meanwhile, microbiota of breast cancer group at 22 dpi presented decreased number of observed features as well as number of nodes and edges compared to its control group (Fig. 3F and G, dotted black lines). Interestingly, when microbiota of control or breast cancer groups were compared over the time, we found that microbiota for both group at 22 dpi presented a higher number of observed features and nodes or edges compared to their counterparts at 16 dpi (Fig. 3F and G, red lines).

**Table 2:** Topological features of the microbial co-occurrence networks.

Topological Features	Experimental groups			
	Control-16dpi	Breast cancer-16dpi	Control-22dpi	Breast cancer-22dpi
Nodes^a^;	678	661	1035	811
Edges^b^	10323	3849	16472	11287
-Positives	5552	2572	9727	7265
-Negatives	4771	1277	7015	4022
Modularity^c^	5.355	1.299	2.506	1.638
Modules^d^	435	770	361	523
Network diameter^e^	8	11	9	9
Average degree^f^	19.259	5.872	25.347	18.016
Weighted degree^g^	1.297	1.639	3.652	4.475
Clustering coefficient^h^	0.531	0.451	0.516	0.522

a Nodes represent bacterial taxa with co-ocurrence correlation SparCC > or < −0.75.

b Edges represent the number of connections/correlations.

c Modularity is the strength of division of a network into modules.

d Modules are sub-communities of bacteria that co-occur more frequently among each other than with other taxa.

e Network diameter is the shortest path between the two most separated nodes.

f Average degree is the average number of links per node.

g Weighted degree is the sum of the weight of all the edges connected to a node.

h Clustering coefficient is the degree to which nodes in a network tend to form clusters.

**Fig. 4:**
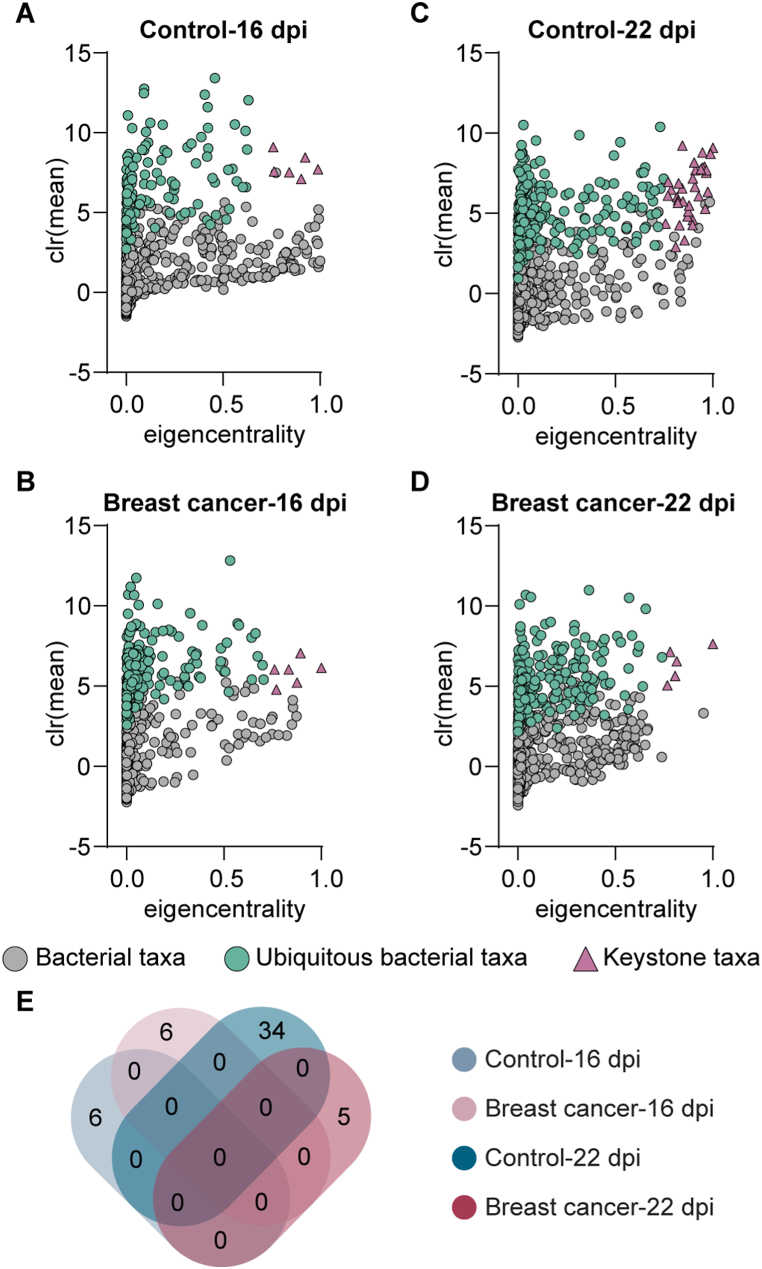
Identification of keystone taxa in the microbial networks of control and breast cancer-bearing mice at different timepoints. Scatter plot of the mean relative abundance, expressed as center log ratio (clr) value vs. the eigenvector centrality of each bacterial taxon (dots or triangle) found in the microbial co-occurrence networks from (A, C) control or (B, D) breast cancer-bearing mice at different timepoints. The green dots or dark red triangles represent ubiquitous bacteria (i.e., taxa that were found across all the samples). Cutoff value of 0.35 were set for the mean relative abundance and 0.75 for the eigenvector centrality. Ubiquitous bacterial taxa with mean relative abundance and eigenvector centrality equal or higher than the cutoff values were considered as keystone taxa (Dark red triangles). (E) Venn diagram shows the number of keystone taxa that are shared by or unique in the different experimental groups. (For interpretation of the references to color in this figure legend, the reader is referred to the Web version of this article.)

To study the hierarchical organization of the microbial community of mice bearing breast cancer, we identified keystone taxa of their microbiota and compared to the control group. We found that the microbiota of breast cancer-bearing mice at 16 dpi (Fig. 4B) presented the same number of keystone taxa to the microbiota of the control group (Fig. 4A). However, at 22 dpi, we found that the microbiota of breast cancer-bearing mice presented 5 keystone taxa (Fig. 4D) which is considerably lower compared to the control group where 34 keystone taxa were identified in its microbiota (Fig. 4C). The list of keystone taxa for each group can be found in Supplementary Table S4. Interestingly, when the identity of the keystone taxa was compared, we found that the keystone taxa were unique for each experimental group (Fig. 4E–Supplementary Table S5). Altogether, these results show that breast cancer produce major shift in the bacterial community assembly and hierarchical organization of mouse microbiota.

### 3.3 Impact of breast cancer on the network robustness

**Fig S1:**
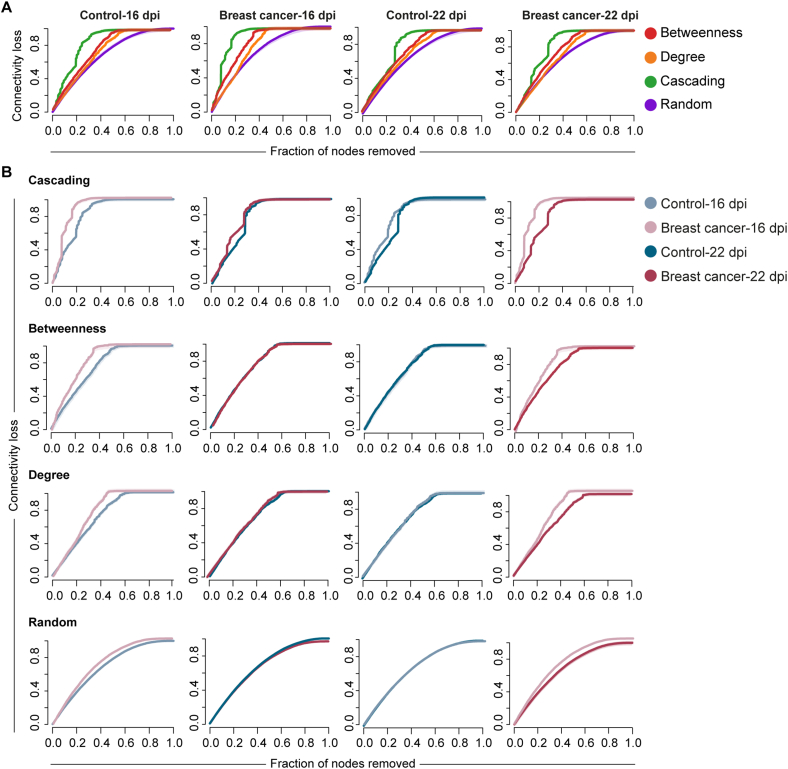
Comparison of network robustness after nodes removal. Connectivity loss measured after directed attack, (betweenness, cascading, degree) or random attack for Control-16 dpi, Breast cancer-16 dpi, Control-22 dpi and Breast cancer-22 dpi (A). The comparison of control *vs* breast cancer-16 dpi, control *vs* breast cancer-22 dpi, control-16 dpi *vs* control-22 dpi and breast cancer-16 dpi *vs* breast cancer-22 dpi networks for cascading, betweenness, degree and random effect, (B).

One crucial aspect of robust networks is their ability to withstand perturbations, such as the removal or addition of nodes. Co-occurrence networks were initially tested for their tolerance to node removal. In this analysis, we measured the resistance of the networks to random or directed removal of nodes and recorded the proportion of taxa removal needed to reach a connectivity loss of 0.80 for each network. It was found that cascading removal had the most significant impact, while random removal had the least impact on all networks, regardless of the presence of tumors in the mice (Supplementary Fig. S1A).

When comparing connectivity loss after node removal using each strategy in cancer-bearing and control mice, we observed that breast cancer-16 dpi had the most deleterious impact on robustness, as a smaller proportion of nodes was needed to reach a connectivity loss of 0.80 in cascading, betweenness, and degree compared to the control group (Supplementary Fig. S1B; Table 3). The network of breast cancer-22 dpi was less robust than control-22 dpi only after cascading removal. Additionally, comparing the robustness in breast cancer-16 dpi and breast cancer-22 dpi revealed major differences between the two, notably breast cancer-16 dpi being less robust than breast cancer-22 dpi (Supplementary Fig. S1B; Table 3). The results suggest that the community's robustness to node removal in cancer-bearing mice increases at 22 dpi compared to 16 dpi, an effect not observed in networks of control mice.

**Table 3:** Fraction of nodes removal required to reach a connectivity loss of 80 %.

Parameters	Control-16 dpi	Breast cancer-16 dpi	Control-22 dpi	Breast cancer-22 dpi
Cascading	0.24	0.16	0.27	0.21
Betweenness	0.39	0.29	0.41	0.31
Degree	0.43	0.35	0.44	0.42
Random	0.51	0.46	0.52	0.49

**Fig S2:**
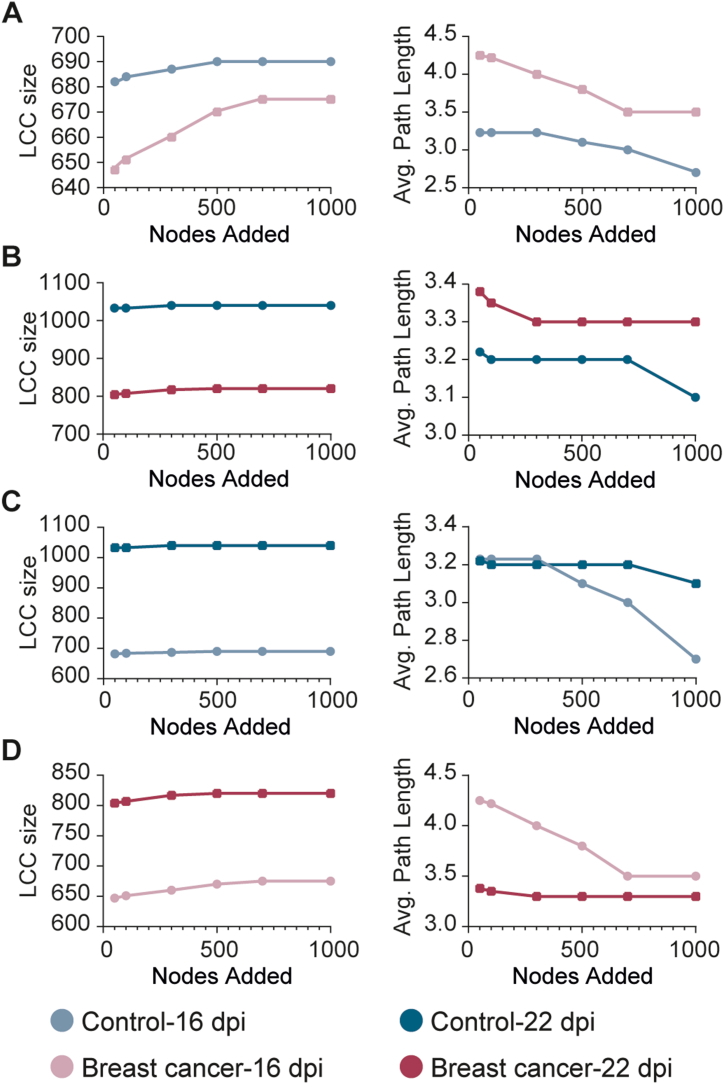
Comparison of network robustness to nodes addition. The value reached of Larges Connected Component size (LCC size) and the average path length for control vs breast cancer-16 dpi (A), control vs breast cancer-22 dpi (B), control-16 dpi vs control-22 dpi (C), breast cancer-16 dpi vs breast cancer-22 dpi (D).

We then tested robustness to taxa addition in all networks. The breast cancer-16 dpi (Supplementary Fig. S2A) and breast cancer-22 dpi (Supplementary Fig. S2B) networks both exhibited smaller LCC compared to their respective control groups. On the other hand, both breast cancer-16 dpi (Supplementary Fig. S2A) and breast cancer-22 dpi networks (Supplementary Fig. S2B) showed a larger APL compared to their respective controls. Small LCC and larger APL are both indicative of no stable networks.

When comparing LCC between the control-16 dpi and control-22 dpi networks (Supplementary Fig. S2C), and between the breast cancer-16 dpi and breast cancer-22 dpi networks (Supplementary Fig. S2D), we observed a larger LCC in breast cancer-22 dpi coincided with a larger LCC in control-22 dpi, compared with control-16 dpi. Indicating that larger LCC in breast cancer-22 dpi compared to breast cancer-16 dpi cannot be associated only to the presence of the tumor.

The APL became shorter with each node addition iteration for both control groups, with a more pronounced decrease in control-16 dpi (Supplementary Fig. S2C), suggesting gains in network robustness with node additions. In contrast, the APL values decreased for breast cancer-16 dpi, while breast cancer-22 dpi maintained relatively stable values along node addition iterations (Supplementary Fig. S2D).

### 3.4 Impact of breast cancer on the predicted metabolic profile of mouse microbiome

**Fig. 5:**
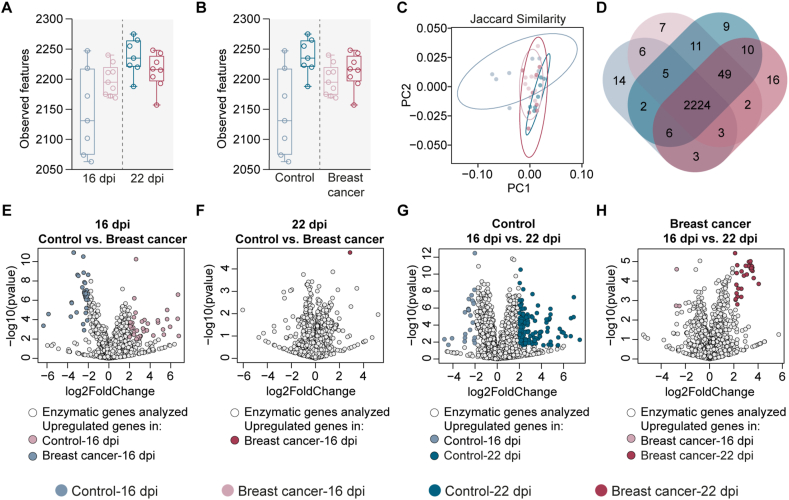
Changes in the enzymatic profile of control and breast cancer-bearing mice at different timepoints. Observed features were used to measure and compare the richness of enzymes between the (A) control and breast cancer-bearing mice at different timepoints or (B) between different timepoints of the same experimental group. (C) Jaccard similarity were used to measure the similarity (PERMANOVA, *p < 0.05*) of the enzymatic profile among the different experimental conditions. (D) Venn diagram showing the number of common and unique enzymes among the enzymatic profile of the microbiota of control or breast cancer-bearing mice at different timepoints. Volcano plot showing the differential enzymatic abundance between the control and breast cancer-bearing mice at (E)16 dpi and (F) 22 dpi and between the (G) control groups or (H) breast cancer-bearing mice. Taxa with significant differences (Wald test, *p < 0.05*) and with log2Fold change >2 or < -2 between the groups are represented with colored dots. The gray dots represent taxa with no significant differences between groups.

**Fig. S3:**
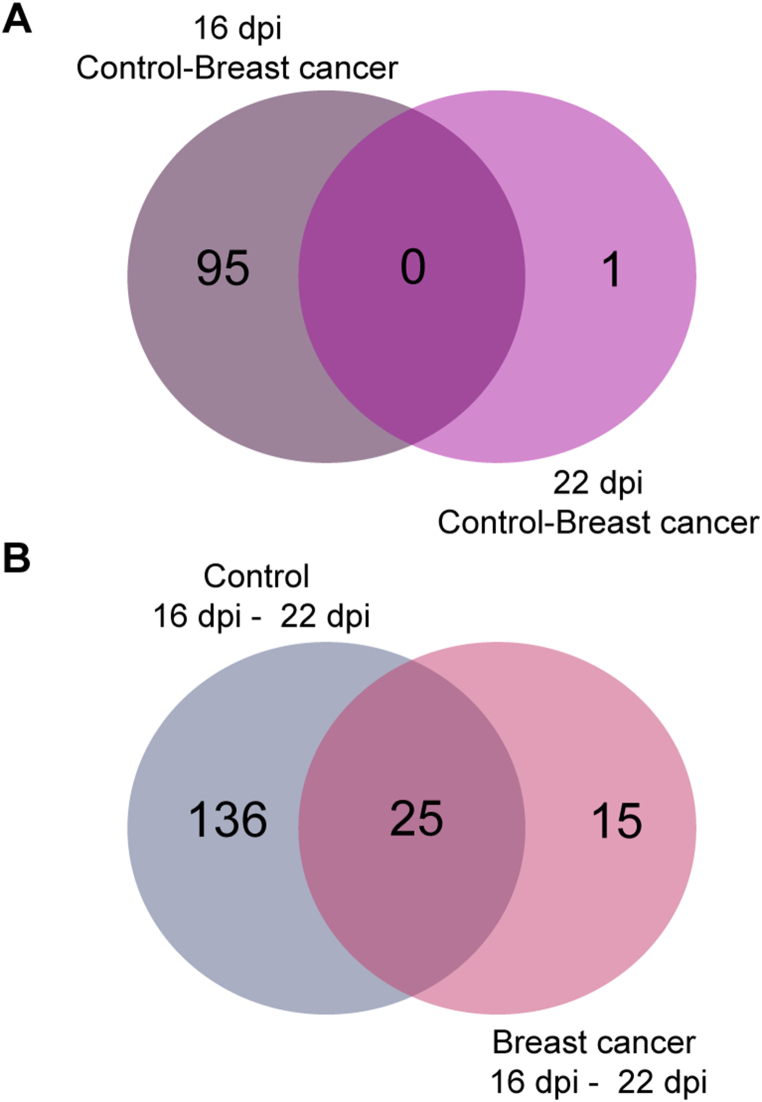
Unique or shared enzymes with differential abundance in control or breast cancer groups at different timepoints. Venn diagram showing the number of common and unique enzymes whose abundance changed significantly between comparisons of (A) Control vs. Breast cancer at 16 dpi and 22 dpi and (B) Control groups at 16 dpi vs 22 dpi and Breast cancer groups at 16 dpi and 22 dpi.

To assess the impact of the exposure to breast cancer cells on the metabolic profile of mouse microbiome, we performed enzyme profiling based on predicted metagenomic functions using PICRUSt2. Comparison of the number of enzymes of the mouse microbiota showed no significant differences between the control and breast cancer groups at either timepoint (Fig. 5A, Kruskal-Wallis, *p > 0.05*). Similarly, significant changes in the number of enzymes were neither found in the control groups nor in the breast cancer groups over the course of time (Fig. 5B, Kruskal-Wallis, *p > 0.05*). Beta diversity analysis revealed a significant difference in the Jaccard similarity index among all the experimental groups (Fig. 5C, PERMANOVA, F = 2.22, *p = 0.002*). Specifically, the metabolic profile of the microbiota of the control group at 16 dpi shows a tendency to separate from the other groups (Fig. 5C). Furthermore, comparison of the metabolic profile showed that the majority of the enzymes (i.e., 2224 of 2367) were shared between the four experimental groups (Fig. 5D–Supplementary Table S6). Further characterization of the changes in the microbiota metabolic profile of breast cancer-bearing mice was accomplished by comparing the abundance of the different enzymes between the different groups. Pairwise comparison between the control and breast cancer-bearing mice microbiota at 16 dpi showed that the abundance of 95 enzymes changed significantly (Fig. 5E, Wald test, *p < 0.05*). In consequence, four pathways were significantly impacted between these groups: aerobactin biosynthesis, reductive TCA cycle II, nitrifier denitrification and sulfoquinovose degradation I pathways (Supplementary Table S7, Wald test, *p < 0.05*). On the other hand, only one enzyme (i.e., EC 6.2.1.2 — Butyrate—CoA ligase) was significantly different in the comparison between the control and breast cancer groups at 22 dpi (Fig. 5F, Wald test, *p < 0.05*). Interestingly, the significant change of this enzyme was exclusively found in the comparison between control and breast cancer groups at 22 dpi but not at 16 dpi (Supplementary Fig. S3A, Supplementary Table S8). We also compared the microbiota of control or breast cancer groups over the time and we found significant changes in the abundance of 161 and 40 enzymes in the microbiome of the control (Fig. 5G, Wald test, *p < 0.05*) or breast cancer (Fig. 5H, Wald test, *p < 0.05*) groups, respectively. The enzymes whose abundance changed significantly were listed in Supplementary Table S9. Furthermore, we found that the pathways that changed significantly between the control groups include amino acid, carbohydrate, nucleotide and secondary metabolite biosynthesis and degradation (Supplementary Table S10, Wald test, *p < 0.05*). Moreover, pathways that changed significantly between the breast cancer groups were essentially those implicated in vitamin and phospholipid biosynthesis (Supplementary Table S11, Wald test, *p < 0.05*).

Comparison of the enzymes with significant changes in their abundance revealed that 25 enzymes were common between the comparisons control-16 dpi vs. control 22 dpi and breast cancer-16 dpi vs. breast cancer-22 dpi (Supplementary Fig. S3B, Supplementary Table S12) while 136 and 15 enzymes were found exclusively in the comparisons control-16 dpi vs. control 22 dpi and breast cancer-16 dpi vs. breast cancer-22 dpi, respectively (Supplementary Fig. S3B, Supplementary Table S12).

**Fig. 6:**
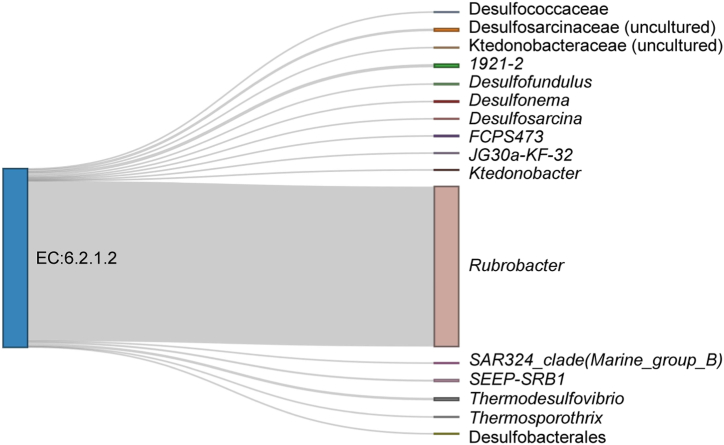
Contribution of bacterial taxa to the enzyme Butyrate--CoA ligase in the microbiome of breast cancer-bearing mice at 22 dpi. Sankey diagram showing the bacterial taxa contributing to the specific enzyme selected. Node segments by columns are showing the enzyme (first column) and bacterial taxa (second column). The size of the node is proportional to the abundance of contributing enzyme or bacterial taxa. The cords represent the connection between the enzymes and taxa. The contribution of each taxon to the enzyme is proportionally represented by the size of the cords.

**Fig. S4:**
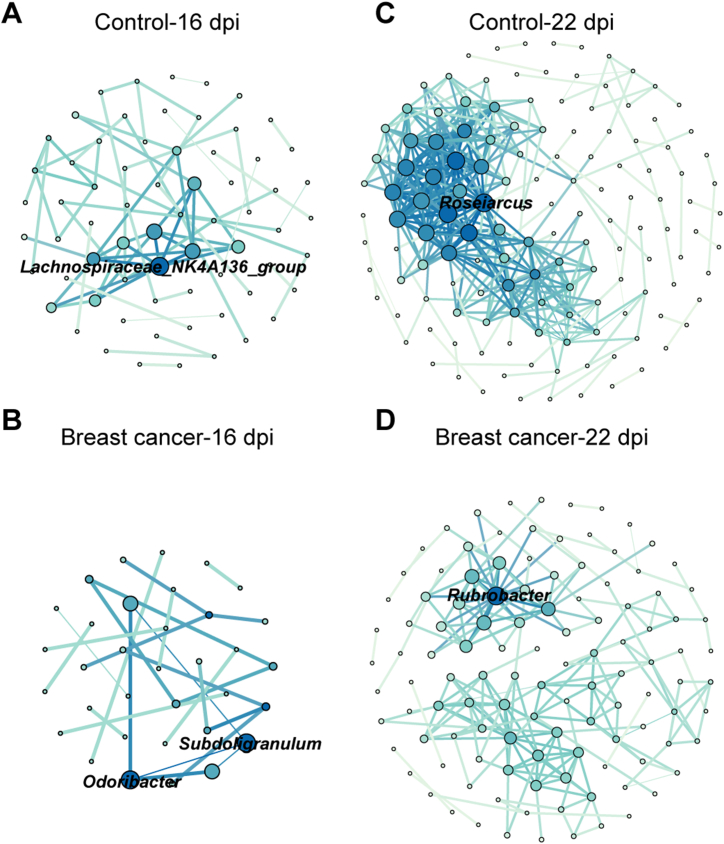
Core co-occurrence microbial networks of control and breast cancer groups at different timepoints. Co-occurrence networks of (A, C) control mice and (B, D) breast cancer-bearing mice at (A, B) 16 dpi and (C, D) 22 dpi are shown. Nodes represent bacterial taxa and edges stand for a co-occurrence correlation (SparCC >0.95 or < −0.95). Node size is proportional to the degree value and node color is based on the eigenvector centrality measure (nodes with darker color has higher eigenvector centrality). Only nodes with at least one connecting edge are displayed. Bacteria with the highest eigenvector centrality are labeled in the core microbial co-occurrence networks.

We next focused in the enzyme EC 6.2.1.2 - Butyrate--CoA ligase and we analyzed the taxa contribution to this particular enzyme (Fig. 6). We found that several taxa of the microbiota of breast cancer-bearing mice contributed to the enzyme. Notably, the taxon with the highest contribution to the Butyrate--CoA ligase enzyme was *Rubrobacter*. This bacterium is the one with the highest eigenvector centrality in the core co-occurrence networks of breast cancer 22 dpi (Supplementary Fig. S4).

## 4 Discussion

The influence of breast cancer on the modulation of the microbiota [[3], [4], [5], [6]], and the increased susceptibility of cancer patients to infectious diseases, including those involving the intestinal tract [23,27,50,51], have been established in previous studies. For successful identification, treatment, and prevention of infections associated with cancer, a comprehensive grasp of predisposing risk factors and common pathogens is essential [52]. In this study, we put forth the hypothesis that microbial community reshaping due to breast cancer may result in a decreased ability of the microbiota to resist colonization. To investigate this phenomenon, we carried out multiple approaches including diversity and composition measures, networks and network robustness tests and prediction of functional traits in the microbiome.

The analysis of alpha diversity indexes revealed that the richness and phylogenetic diversity did not differ significantly between the control mice and breast cancer-exposed mice at either 16 or 22 dpi. However, both groups showed significant changes in the diversity of their microbiota over time, suggesting that temporal factors play a role in shaping the gut microbiome in both conditions. While these findings contrast with previous reports, where breast cancer was associated with lower [53,54] or higher [55] microbiota diversity, they are consistent with other studies where no significant differences in alpha diversity between cancer patients and healthy individuals were found [[56], [57], [58]]. These discrepancies may arise from differences in target species, experimental design, or methodologies. It is essential to consider that breast cancer is a heterogeneous disease with various subtypes and stages [59,60], and its interaction with the gut microbiota may vary depending on these factors. Reduced colonization resistance has been linked to a decrease in diversity of microbial species within the microbiota in other systems [61,62]. Thus, when examined independently, our findings on alpha diversity suggest that breast cancer has a minimal impact on colonization resistance.

However, the beta diversity analysis revealed a shift in the bacterial community composition and abundance in breast cancer-exposed mice compared to control mice, particularly at 16 dpi. This indicates that breast cancer exposure has an immediate impact on the gut microbiome's structure as previously reported [[53], [54], [55]], potentially altering its ability to resist colonization by pathogenic microorganisms. Notably, the differences in beta diversity between the two groups diminished at 22 dpi, suggesting a possible stabilization or adaptation of the microbiota in response to breast cancer over time. These findings suggest that while the overall diversity of the microbiota may not be impacted, specific microbial communities crucial for colonization resistance could be compromised by the presence of the tumor.

Network analysis revealed that breast cancer exposure led to a shift in bacterial community assembly patterns, and the networks in breast cancer-bearing mice exhibited a decreased number of nodes and edges compared to the control groups. The decreased number of edges and modularity in the networks of breast cancer-bearing mice suggests a disruption in microbial interactions and communication, as described in other systems [63,64]. Furthermore, the impact of breast cancer on colonization resistance was evaluated through a comprehensive analysis of network robustness using three distinct tests. Firstly, when assessing the vulnerability of network connectivity to node removal, breast cancer-bearing mice at 16 dpi displayed decreased robustness compared to control mice, indicating a higher susceptibility to connectivity loss. Interestingly, at 22 dpi, the breast cancer network exhibited improved robustness, suggesting a temporal increase in resistance to connectivity disruption. Secondly, in the context of adding new nodes, both breast cancer-16 dpi and breast cancer-22 dpi networks exhibited lower LCC values compared to their respective control groups, indicating potential challenges in maintaining cohesive network structures. Moreover, these cancer-bearing networks demonstrated higher APL values, implying reduced efficiency in information flow. These contrasting APL responses to node addition highlight the complexity of information dissemination within cancer-bearing networks. Overall, the results suggest that breast cancer can compromise colonization resistance by influencing network connectivity, structure, and efficiency, with temporal dynamics further influencing the interplay between disease and microbial interactions.

The disruption of community assembly in the microbiota, which can weaken its stability and resistance to pathogen colonization, is a phenomenon shared between non-cancer disturbing factors and cancer itself. Various factors such as drug administration (e.g., anthelmintics, antibiotics) [65,66], pathogen infections [67], and chemotherapy have been shown to disrupt the balance between the host and the microbiota. For instance, anthelmintic drugs and antibiotics have been associated with altering microbial communities, destabilizing interactions, and compromising resilience [65]. In the case of antibiotics, disruption of the microbiota's balance can lead to infections like *Clostridium difficile*-associated colitis [66], highlighting the importance of colonization resistance in preventing pathogenic overgrowth. This disruption in microbial stability is also seen in cancer patients, particularly those with solid tumors, who face infections involving the intestinal tract [23]. Neutropenic enterocolitis (NEC), once primarily associated with acute leukemia patients, has been observed in solid tumor patients receiving chemotherapy [[68], [69], [70], [71]]. This condition is characterized by abdominal symptoms, fever, and diarrhea, resembling the symptoms of other intestinal infections. Similarly, risk factors associated with *C. difficile* colonization and disease includes antineoplastic chemotherapy, antibiotic usage, and proton-pump inhibitors [72,73]. These findings suggest that reduced colonization resistance due to both cancer and non-cancer factors can lead to similar effects, compromising the microbiota's resilience and fostering susceptibility to infections, particularly those involving the intestinal tract. Consequently, interventions to maintain colonization resistance become essential in both cancer treatment and general healthcare contexts.

The assessment of the predicted metabolic profile of the mouse microbiome revealed no significant differences in the number of enzymes between control and breast cancer groups at either time point. However, significant changes in the abundance of enzymes were observed in the comparison of control-16 dpi vs. control-22 dpi and breast cancer-16 dpi vs. breast cancer-22 dpi. Notably, the enzyme EC 6.2.1.2 - Butyrate--CoA ligase showed significant increase in abundance in the microbiome of breast cancer-bearing mice at 22 dpi. Butyrate--CoA ligase participates in the metabolism of butyrate, a well-known molecule in microbiota-cancer interactions, as along with propionate and acetate, it is a tumor suppressor for a myriad of different cancer types, especially colon cancer [74]. Butyrate produced by gut microorganisms regulates gene expression and effectively protects intestinal epithelial cells from damage and preventing the development of colon cancer [75], and relieving pathogenic bacterial-caused acute inflammation [76]. Various studies have indicated that butyrate limits the proliferation of breast cancer cell lines by functioning as an histone deacetylase inhibitors and stimulating the formation of cyclin-dependent kinase inhibitor p21 [[77], [78], [79]]. Butyrate is also negatively associated with *C. difficile* burdens in humans and mice and this molecule impedes the growth of diverse *C. difficile* strains in pure culture [80,81]. Thus, an eventual increase of butyrate in the context of breast cancer could balance the disruption of community assembly in the microbiota and provide colonization resistance to some pathobionts. However, this needs to be validated by further experimentation.

Interestingly, the Butyrate--CoA ligase was associated mainly with the taxon *Rubrobacter*, an aerobic keystone species in the breast cancer-22 dpi network. Conversely, other study has associated the production of butyrate with anaerobic bacteria such as *Coprococcus* and *Dorea*, both found in the gut microbiota of breast cancer patients [82]. We found *Coprococcus* as a keystone bacterium in the breast cancer-16 dpi network but not at 22 dpi. This suggests that as gut microbiota adapts to breast cancer over time, significant changes in the gut environment may occur. These changes might not favor the development of certain anaerobic bacteria, but instead promote the growth of aerobic bacteria such as *Rubrobacter*, which could then play a central role in the gut microbiota. Furthermore, Rubrobacter has been found as a core bacterium in bladder cancer, with significantly higher abundance than in control samples [83]. *Rubrobacter* was also found among the top 5 most abundant bacterial genera in the microbiota of breast tissue in breast cancer patients [84]. Loss or changes of keystone taxa in breast tumors could disrupt homeostatic microbiome–immune interactions [18], leading to immune dysregulation, carcinogenesis [85,86], and infection [87]. This suggests a potential link between specific bacterial taxa, enzyme activities, and the metabolic response of the gut microbiota to breast cancer exposure.

Commensal bacteria, traditionally seen as benign constituents of the microbiota, can adopt an opportunistic pathogenic behavior in response to the altered physiological conditions induced by breast cancer [88,89]. This shift can critically influence both the microbiota's resilience to external pathogens and the host's overall disease susceptibility. Factors contributing to this opportunistic transformation include pathoadaptive mutations [90], alterations in the availability of preferred nutrients [89], and strategies to evade the host immune response [91]. These mechanisms are pivotal in understanding the complex interplay between breast cancer and microbiota dynamics, as they underline the potential for commensals to exacerbate disease states or influence the efficacy of cancer treatments.

Specifically, pathoadaptive mutations allow commensals to acquire virulence traits [90], enabling them to exploit the compromised mucosal barrier and immune dysregulation commonly associated with cancer [92]. Moreover, the cancer-altered gut environment can lead to shifts in nutrient availability [93], favoring the overgrowth of certain commensals that can outcompete beneficial microbes and exacerbate disease processes [93,94]. Furthermore, the ability of these commensals to sidestep immune surveillance can lead to persistent inflammation and tissue damage [91], contributing to a pro-carcinogenic environment.

Our findings on the microbiome's structural changes and functional traits in breast cancer models suggest that the disease may indeed facilitate an environment that promotes the opportunistic behavior of commensals. Notably, the increased abundance of enzymes like Butyrate--CoA ligase, associated with beneficial metabolic activities, also hints at the complex relationship between metabolic pathways, microbial community structure, and cancer progression. The observed shifts in microbial community assembly and network properties further support the hypothesis that breast cancer can alter the gut ecosystem in a manner that compromises its stability and resistance to pathogen colonization, potentially through the opportunistic actions of commensal bacteria.

## 5 Conclusions

In conclusion, our study provides insights into the complex interplay between breast cancer and the gut microbiome, particularly focusing on colonization resistance. We demonstrated that breast cancer exposure can lead to alterations in the microbial community structure, organization, and stability. Breast cancer exposure can also alter the metabolic profile of the gut microbiota by increasing the expression of butyrate--CoA ligase and potentially balancing the reduction of colonization resistance. These changes may impact the ability of the microbiota to resist colonization by pathogenic microorganisms. Our findings highlight the importance of considering the role of the gut microbiome in breast cancer progression and the potential implications for therapeutic interventions and preventive measures. Further studies are needed to elucidate the underlying mechanisms and to validate the potential of modulation of the gut microbiome as a strategy to enhance colonization resistance and improve outcomes for breast cancer patients.

## Data availability statement

The raw 16S rRNA gene sequences obtained from mouse feces samples were deposited at the SRA repository (Bioproject No. PRJNA1008984).

## Ethics statement

All animal studies were performed at the Animal Facility of the National Center for Laboratory Animal Breeding (CENPALAB), Havana, Cuba, according to National and International Guiding Principles for Biomedical Research Involving Animals. All procedures were reviewed and approved by the Institutional Animal Care and Use Committee (Ethics Committee) of CENPALAB, with permit number 24/22.

## CRediT authorship contribution statement

**Alejandra Wu-Chuang:** Writing – review & editing, Writing – original draft, Visualization, Methodology, Formal analysis. **Lourdes Mateos-Hernandez:** Writing – review & editing, Formal analysis. **Lianet Abuin-Denis:** Writing – original draft, Methodology, Formal analysis. **Apolline Maitre:** Writing – review & editing, Formal analysis. **Janet Avellanet:** Writing – review & editing, Methodology, Formal analysis. **Arlem García:** Writing – review & editing, Methodology, Formal analysis. **Dasha Fuentes:** Writing – review & editing, Resources, Methodology, Formal analysis, Conceptualization. **Alejandro Cabezas-Cruz:** Writing – review & editing, Writing – original draft, Supervision, Resources, Conceptualization.

## Declaration of competing interest

The authors declare that they have no known competing financial interests or personal relationships that could have appeared to influence the work reported in this paper.

## Appendix Supplementary data

Multimedia component 1 Bacterial taxa found as contaminants in the 16S rRNA gene sequencing datasets from control or breast cancer-bearing mice. Contaminants were statistically identified (TRUE) and removed from the 16S rRNA gene sequencing datasets using the decontam R package. Multimedia component 1

Multimedia component 2 Unique or shared bacterial taxa of control or breast cancer groups at different time points. Multimedia component 2

Multimedia component 3 Unique or shared nodes in microbial co-occurrence networks of control or breast cancer groups at different time points. Multimedia component 3

Multimedia component 4 List of keystone taxa in the microbial co-occurrence networks of control or breast cancer groups at different time points. Multimedia component 4

Multimedia component 5 Unique or shared keystone taxa in microbial co-occurrence networks of control or breast cancer groups at different time points. Multimedia component 5

Multimedia component 6 Unique or shared predicted enzymes in the metabolic profile of control or breast cancer groups at different time points. Multimedia component 6

Multimedia component 7 Pathway with differential abundance in the microbiota of control or breast cancer groups at 16 dpi. Multimedia component 7

Multimedia component 8 Unique or shared enzymes with differential abundance between the control and breast cancer groups at different timepoints. Multimedia component 8

Multimedia component 9 Enzymes with differential abundance in the microbiota of control or breast cancer groups at different time points. Multimedia component 9

Multimedia component 10 Pathway with differential abundance in the microbiota of control groups at different timepoints. Multimedia component 10

Multimedia component 11 Pathway with differential abundance in the microbiota of breast cancer groups at different timepoints. Multimedia component 11

Multimedia component 12 Unique or shared enzymes with differential abundance between the control or breast cancer groups at different timepoints. Multimedia component 12

## References

[1] Wilkinson L., Gathani T. (2022). Understanding breast cancer as a global health concern. Br. J. Radiol..

[2] Sadrekarimi H., Gardanova Z.R., Bakhshesh M. (2022). Emerging role of human microbiome in cancer development and response to therapy: special focus on intestinal microflora. J. Transl. Med..

[3] Banerjee S., Tian T., Wei Z. (2018). Distinct microbial signatures associated with different breast cancer types. Front. Microbiol..

[4] Yang P., Wang Z., Peng Q., Lian W., Chen D. (2021). Comparison of the gut microbiota in patients with benign and malignant breast tumors: a pilot study. Evol Bioinform Online.

[5] Xuan C., Shamonki J.M., Chung A. (2014). Microbial dysbiosis is associated with human breast cancer. PLoS One.

[6] Álvarez-Mercado A.I., del Valle Cano A., Fernández M.F., Fontana L. (2023). Gut microbiota and breast cancer: the dual role of microbes. Cancers.

[7] Dey P., Chaudhuri S.R. (2022). Cancer-associated microbiota: from mechanisms of disease Causation to microbiota-centric anti-cancer approaches. Biology.

[8] Wu-Chuang A., Mateos-Hernandez L., Maitre A. (2023). Microbiota perturbation by anti-microbiota vaccine reduces the colonization of *Borrelia afzelii* in *Ixodes ricinus*. Microbiome.

[9] Maitre A., Wu-Chuang A., Mateos-Hernández L. (2023). Rickettsial pathogens drive microbiota assembly in *Hyalomma marginatum* and *Rhipicephalus bursa* ticks. Mol. Ecol..

[10] Corduneanu A., Wu-Chuang A., Maitre A., Obregon D., Sándor A.D., Cabezas-Cruz A. (2023). Structural differences in the gut microbiome of bats using terrestrial vs. aquatic feeding resources. BMC Microbiol..

[11] Svobodová K., Maitre A., Obregón D. (2023). Gut microbiota assembly of Gotland varroa-surviving honey bees excludes major viral pathogens. Microbiol. Res..

[12] Lee W.H., Chen H.M., Yang S.F. (2017). Bacterial alterations in salivary microbiota and their association in oral cancer. Sci. Rep..

[13] Najafi S., Jamalkandi S.A., Najafi A., Salimian J., Ahmadi A. (2023). Exploring Co-occurrence patterns and microbial diversity in the lung microbiome of patients with non-small cell lung cancer. BMC Microbiol..

[14] Banerjee S., Schlaeppi K., van der Heijden M.G.A. (2018). Keystone taxa as drivers of microbiome structure and functioning. Nat. Rev. Microbiol..

[15] Wu-Chuang A., Bates K.A., Obregon D., Estrada-Peña A., King K.C., Cabezas-Cruz A. (2022). Rapid evolution of a novel protective symbiont into keystone taxon in *Caenorhabditis elegans* microbiota. Sci. Rep..

[16] Wu-Chuang A., Obregon D., Estrada-Peña A., Cabezas-Cruz A. (2021). Thermostable keystone bacteria maintain the functional diversity of the *Ixodes scapularis* microbiome under heat stress. Microb. Ecol..

[17] Liu Y., Li X., Yang Y. (2021). Exploring gut microbiota in patients with colorectal disease based on 16S rRNA gene amplicon and shallow metagenomic sequencing. Front. Mol. Biosci..

[18] Tzeng A., Sangwan N., Jia M. (2021). Human breast microbiome correlates with prognostic features and immunological signatures in breast cancer. Genome Med..

[19] Stacy A., Andrade-Oliveira V., McCulloch J.A. (2021). Infection trains the host for microbiota-enhanced resistance to pathogens. Cell.

[20] Karita Y., Limmer D.T., Hallatschek O. (2022). Scale-dependent tipping points of bacterial colonization resistance. Proc Natl Acad Sci U S A.

[21] Ducarmon Q.R., Zwittink R.D., Hornung B.V.H., van Schaik W., Young V.B., Kuijper E.J. (2019). Gut microbiota and colonization resistance against bacterial enteric infection. Microbiol. Mol. Biol. Rev..

[22] Mullineaux-Sanders C., Suez J., Elinav E., Frankel G. (2018). Sieving through gut models of colonization resistance. Nat Microbiol.

[23] Rolston K.V.I. (2017). Infections in cancer patients with solid tumors: a review. Infect. Dis. Ther..

[24] Rasool Hassan B.A., Yusoff Z.B.M., Othman S.B. (2010). Fever/clinical signs and association with neutropenia in solid cancer patients--bacterial infection as the main cause. Asian Pac. J. Cancer Prev. APJCP.

[25] Kim Y.I.l., Kang H.C., Lee H.S. (2014). Invasive pulmonary mucormycosis with concomitant lung cancer presented with massive hemoptysis by huge pseudoaneurysm of pulmonary artery. Ann. Thorac. Surg..

[26] Carr S.B., Adderson E.E., Hakim H., Xiong X., Yan X., Caniza M. (2012). Clinical and demographic characteristics of seasonal influenza in pediatric patients with cancer. Pediatr. Infect. Dis. J..

[27] Zorina T., Styche A. (2015).

[28] Mateos-Hernández L., Obregon D., Wu-Chuang A. (2021). Anti-microbiota vaccines modulate the tick microbiome in a taxon-specific manner. Front. Immunol..

[29] Freitas S., Yang D., Kumar S., Tong H., Chau D.H. (2020). Evaluating graph vulnerability and robustness using TIGER. Int Conf Inf Knowl Manag Proc.

[30] Parada A.E., Needham D.M., Fuhrman J.A. (2016). Every base matters: assessing small subunit rRNA primers for marine microbiomes with mock communities, time series and global field samples. Environ. Microbiol..

[31] Apprill A., Mcnally S., Parsons R., Weber L. (2015). Minor revision to V4 region SSU rRNA 806R gene primer greatly increases detection of SAR11 bacterioplankton. Aquat. Microb. Ecol..

[32] Davis N.M., Proctor D.M., Holmes S.P., Relman D.A., Callahan B.J. (2018). Simple statistical identification and removal of contaminant sequences in marker-gene and metagenomics data. Microbiome.

[33] Bolyen E., Rideout J.R., Dillon M.R. (2019). Reproducible, interactive, scalable and extensible microbiome data science using QIIME 2. Nat. Biotechnol..

[34] Callahan B.J., McMurdie P.J., Rosen M.J., Han A.W., Johnson A.J.A., Holmes S.P. (2016). DADA2: high-resolution sample inference from Illumina amplicon data. Nat. Methods.

[35] Bokulich N.A., Kaehler B.D., Rideout J.R. (2018). Optimizing taxonomic classification of marker-gene amplicon sequences with QIIME 2's q2-feature-classifier plugin. Microbiome.

[36] Yarza P., Yilmaz P., Pruesse E. (2014). Uniting the classification of cultured and uncultured bacteria and archaea using 16S rRNA gene sequences. Nat. Rev. Microbiol..

[37] Friedman J., Alm E.J. (2012). Inferring correlation networks from genomic survey data. PLoS Comput. Biol..

[38] (2020). RStudio Team, RStudio.

[39] Bastian M., Heymann S., Jacomy M. (2009). Third Int AAAI Conf Weblogs Soc Media.

[40] Ruhnau B. (2000). Eigenvector-centrality—a node-centrality?. Soc. Network..

[41] NetSwan-package: Network Strengths and Weaknesses Analysis in NetSwan: Network Strengths and Weaknesses Analysis.

[42] Douglas G.M., Maffei V.J., Zaneveld J.R. (2020). PICRUSt2 for prediction of metagenome functions. Nat. Biotechnol..

[43] Kanehisa M., Goto S S. (2000). KEGG: kyoto Encyclopedia of genes and Genomes. Nucleic Acids Res..

[44] Tatusov R.L., Galperin M.Y., Natale D.A., Koonin E.V. (2000). The COG database: a tool for genome-scale analysis of protein functions and evolution. Nucleic Acids Res..

[45] Caspi R., Billington R., Fulcher C.A. (2018). The MetaCyc database of metabolic pathways and enzymes. Nucleic Acids Res..

[46] Fernandes A.D., Reid J.N., Macklaim J.M., McMurrough T.A., Edgell D.R., Gloor G.B. (2014). Unifying the analysis of high-throughput sequencing datasets: characterizing RNA-seq, 16S rRNA gene sequencing and selective growth experiments by compositional data analysis. Microbiome.

[47] Love M.I., Huber W., Anders S. (2014). Moderated estimation of fold change and dispersion for RNA-seq data with DESeq2. Genome Biol..

[48] Lex A., Gehlenborg N., Strobelt H., Vuillemot R., Pfister H. (2014). UpSet: visualization of intersecting sets. IEEE Trans. Vis. Comput. Graph..

[49] Conway J.R., Lex A., Gehlenborg N N. (2017). UpSetR: an R package for the visualization of intersecting sets and their properties. Bioinformatics.

[50] Mu X.M., Wang W., Wu F.Y., Jiang Y.Y., Ma L.L., Feng J. (2020). Comorbidity in older patients hospitalized with cancer in Northeast China based on hospital discharge data. Int. J. Environ. Res. Publ. Health.

[51] Stosor V., Zembower T.R. (2020).

[52] Zembower T.R. (2014). Epidemiology of infections in cancer patients. Infect Complicat Cancer Patients.

[53] Goedert J.J., Hua X., Bielecka A. (2018). Postmenopausal breast cancer and oestrogen associations with the IgA-coated and IgA-noncoated faecal microbiota. Br. J. Cancer.

[54] Bobin-Dubigeon C., Luu H.T., Leuillet S. (2021). Faecal microbiota composition varies between patients with breast cancer and healthy women: a comparative case-control study. Nutrients.

[55] Zhu J., Liao M., Yao Z. (2018). Breast cancer in postmenopausal women is associated with an altered gut metagenome. Microbiome.

[56] Jones G.S., Feigelson H.S., Falk R.T. (2019). Mammographic breast density and its association with urinary estrogens and the fecal microbiota in postmenopausal women. PLoS One.

[57] Wu A.H., Tseng C., Vigen C. (2020). Gut microbiome associations with breast cancer risk factors and tumor characteristics: a pilot study. Breast Cancer Res. Treat..

[58] Wang H., Altemus J., Niazi F. (2017). Breast tissue, oral and urinary microbiomes in breast cancer. Oncotarget.

[59] Guo L., Kong D., Liu J. (2023). Breast cancer heterogeneity and its implication in personalized precision therapy. Exp. Hematol. Oncol..

[60] Testa U., Castelli G., Pelosi E E. (2020). Breast cancer: a molecularly heterogenous disease needing subtype-specific treatments. Med. Sci..

[61] Robinson C.J., Schloss P., Ramos Y., Raffa K., Handelsman J. (2010). Robustness of the bacterial community in the cabbage white butterfly larval midgut. Microb. Ecol..

[62] Shea K., Chesson P. (2002). Community ecology theory as a framework for biological invasions. Trends Ecol. Evol..

[63] Gao C., Xu L., Montoya L. (2022). Co-occurrence networks reveal more complexity than community composition in resistance and resilience of microbial communities. Nat. Commun..

[64] Baldassano S.N., Bassett D.S. (2016). Topological distortion and reorganized modular structure of gut microbial co-occurrence networks in inflammatory bowel disease. Sci. Rep..

[65] Boisseau M., Dhorne-Pollet S., Bars-Cortina D. (2023). Species interactions, stability, and resilience of the gut microbiota - helminth assemblage in horses. iScience.

[66] Britton R.A., Young V.B. (2012). Interaction between the intestinal microbiota and host in Clostridium difficile colonization resistance. Trends Microbiol..

[67] Kamdar K., Khakpour S., Chen J. (2016). Genetic and metabolic signals during acute enteric bacterial infection alter the microbiota and drive progression to chronic inflammatory disease. Cell Host Microbe.

[68] Gorschlüter M., Marklein G., Höfling K. (2002). Abdominal infections in patients with acute leukaemia: a prospective study applying ultrasonography and microbiology. Br. J. Haematol..

[69] Capria S., Vitolo D., Cartoni C. (2004). Neutropenic enterocolitis in acute leukemia: diagnostic and therapeutic dilemma. Ann. Hematol..

[70] Snydman D.R., Nesher L., Rolston K.V.I. (2013). Neutropenic enterocolitis, a growing concern in the era of widespread use of aggressive chemotherapy. Clin. Infect. Dis..

[71] Ibrahim N.K., Sahin A.A., Dubrow R.A. (2000). Colitis associated with docetaxel-based chemotherapy in patients with metastatic breast cancer. Lancet (London, England).

[72] Loo V.G., Bourgault A.-M., Poirier L. (2011). Host and pathogen factors for Clostridium difficile infection and colonization. N. Engl. J. Med..

[73] Emoto M., Kawarabayashi T., Hachisuga T., Eguchi F., Shirakawa K. (1996). Clostridium difficile colitis associated with cisplatin-based chemotherapy in ovarian cancer patients. Gynecol. Oncol..

[74] Yang Q., Wang B., Zheng Q. (2023). A review of gut microbiota-derived metabolites in tumor progression and cancer therapy. Adv. Sci..

[75] Mathewson N.D., Jenq R., Mathew A.V. (2016). Gut microbiome-derived metabolites modulate intestinal epithelial cell damage and mitigate graft-versus-host disease. Nat. Immunol..

[76] Chen J., Vitetta L. (2020). The role of butyrate in attenuating pathobiont-induced hyperinflammation. Immune Netw.

[77] De Los Santos M., Martínez-Iglesias O., Aranda A. (2007). Anti-estrogenic actions of histone deacetylase inhibitors in MCF-7 breast cancer cells. Endocr. Relat. Cancer.

[78] Walker G.E., Wilson E.M., Powell D., Oh Y. (2001). Butyrate, a histone deacetylase inhibitor, activates the human IGF binding protein-3 promoter in breast cancer cells: molecular mechanism involves an Sp1/Sp3 multiprotein complex. Endocrinology.

[79] Chopin V., Toillon R.A., Jouy N., Le Bourhis X. (2004). P21(WAF1/CIP1) is dispensable for G1 arrest, but indispensable for apoptosis induced by sodium butyrate in MCF-7 breast cancer cells. Oncogene.

[80] Pensinger D.A., Fisher A.T., Dobrila H.A. (2023). Butyrate differentiates permissiveness to clostridioides difficile infection and influences growth of diverse C. difficile isolates. Infect. Immun..

[81] Wang S., Xiang L., Li F., Deng W., lv P., Chen Y. (2023). Butyrate protects against Clostridium difficile infection by regulating bile acid metabolism. Microbiol. Spectr..

[82] Li Y., Dong B., Wu W. (2022). Metagenomic analyses reveal distinct gut microbiota signature for predicting the neoadjuvant chemotherapy responsiveness in breast cancer patients. Front. Oncol..

[83] Mai G., Chen L., Li R., Liu Q., Zhang H., Ma Y. (2019). Common core bacterial biomarkers of bladder cancer based on multiple datasets. BioMed Res. Int..

[84] Thyagarajan S., Zhang Y., Thapa S. (2020). Comparative analysis of racial differences in breast tumor microbiome. Sci. Rep..

[85] Peterson C.T., Sharma V., Elmén L., Peterson S.N. (2015). Immune homeostasis, dysbiosis and therapeutic modulation of the gut microbiota. Clin. Exp. Immunol..

[86] Riquelme E., Zhang Y., Zhang L. (2019). Tumor microbiome diversity and composition influence pancreatic cancer outcomes. Cell.

[87] Zheng D., Liwinski T., Elinav E. (2020). Interaction between microbiota and immunity in health and disease. Cell Res..

[88] Dey P., Ray Chaudhuri S. (2023). The opportunistic nature of gut commensal microbiota. Crit. Rev. Microbiol..

[89] Dey P. (2024). Good girl goes bad: understanding how gut commensals cause disease. Microb. Pathog..

[90] Sokurenko E.V., Hasty D.L., Dykhuizen D.E. (1999). Pathoadaptive mutations: gene loss and variation in bacterial pathogens. Trends Microbiol..

[91] Mues N., Chu H.W. (2020). Out-smarting the host: bacteria maneuvering the immune response to favor their survival. Front. Immunol..

[92] Genua F., Raghunathan V., Jenab M., Gallagher W.M., Hughes D.J. (2021). The role of gut barrier dysfunction and microbiome dysbiosis in colorectal cancer development. Front. Oncol..

[93] Benešová I., Křížová Ľ., Kverka M. (2023). Microbiota as the unifying factor behind the hallmarks of cancer. J. Cancer Res. Clin. Oncol..

[94] Ivleva E.A., Grivennikov S.I. (2022). Microbiota-driven mechanisms at different stages of cancer development. Neoplasia.

